# Tailoring Potential Natural Compounds for the Treatment of Luminal Breast Cancer

**DOI:** 10.3390/ph16101466

**Published:** 2023-10-16

**Authors:** Fernanda Cardoso da Silva, Douglas Cardoso Brandão, Everton Allan Ferreira, Raoni Pais Siqueira, Helen Soares Valença Ferreira, Ademar Alves Da Silva Filho, Thaise Gonçalves Araújo

**Affiliations:** 1 Laboratory of Genetics and Biotechnology, Institute of Biotechnology, Universidade Federal de Uberlândia, Patos de Minas 38700-002, MG, Brazil; fernanda.cardoso95@yahoo.com (F.C.d.S.); douglasbrandao10@hotmail.com (D.C.B.); raoni.siqueira@ufu.br (R.P.S.); helensvalenca@gmail.com (H.S.V.F.); 2 Department of Pharmaceutical Sciences, Faculty of Pharmacy, Federal University of Juiz de Fora, Juiz de Fora 36036-900, MG, Brazil; everton.allan888@gmail.com (E.A.F.); ademar.alves@ufjf.br (A.A.D.S.F.); 3 Laboratory of Nanobiotechnology Prof. Dr. Luiz Ricardo Goulart Filho, Institute of Biotechnology, Universidade Federal de Uberlândia, Uberlandia 38405-302, MG, Brazil

**Keywords:** breast cancer, hormone therapy, natural products, resistance

## Abstract

Breast cancer (BC) is the most diagnosed cancer worldwide, mainly affecting the epithelial cells from the mammary glands. When it expresses the estrogen receptor (ER), the tumor is called luminal BC, which is eligible for endocrine therapy with hormone signaling blockade. Hormone therapy is essential for the survival of patients, but therapeutic resistance has been shown to be worrying, significantly compromising the prognosis. In this context, the need to explore new compounds emerges, especially compounds of plant origin, since they are biologically active and particularly promising. Natural products are being continuously screened for treating cancer due to their chemical diversity, reduced toxicity, lower side effects, and low price. This review summarizes natural compounds for the treatment of luminal BC, emphasizing the activities of these compounds in ER-positive cells. Moreover, their potential as an alternative to endocrine resistance is explored, opening new opportunities for the design of optimized therapies.

## 1. Introduction

Epidemiological data on cancer are alarming. According to the World Health Organization (WHO), in 2020, more than 19 million new cases of the disease and approximately 10 million associated deaths were recorded worldwide. In 2023, 1,958,310 new cancer cases and 609,820 cancer deaths are expected in the United States. Breast cancer (BC) has the highest incidence rate of all cancers, accounting for 2.3 million diagnoses in 2020. Most of the new cases and disease-related mortality from BC occur in low- and middle-income countries. In high-income countries, the chance of survival exceeds 80%, in contrast to developing countries where the diagnosis still occurs late [1,2]. In addition, prognostic factors such as tumor size, grade, lymph node involvement, and estrogen receptor (ER) expression are essential in choosing the therapeutic strategy. In fact, BC is classified molecularly according to the expression of human epidermal growth factor receptor 2 (HER2), progesterone receptor, and ER [3].

ER-positive tumors are defined as luminal, account for about two-thirds of cases, and show an intrinsic heterogeneity from the histological, transcriptional, and mutational points of view, with different clinical courses and therapeutic strategies [4]. Although patients with luminal BC have a better prognosis, in 30% of these cases there is late recurrence of the disease (after 5 to 10 years), mainly at a distance, with a predominance of bone metastases. This scenario directly impacts the data on the overall survival, and the risk of recurrence for patients with luminal BC is real. The time and characteristics of this progression are affected not only by the prognosis, but also by the adjuvant therapeutic strategies adopted [5].

For ER-positive tumors, surgery, radiotherapy, chemotherapy, targeted therapy, and hormone therapy are recommended as established methods. However, different questions are still raised in clinical practice: Which patients really benefit from chemotherapy? How to reverse endocrine resistance? What are the challenges in the development of new drugs for the treatment of luminal BC? In this context, natural compounds have shown to be potentially promising.

Plants produce secondary metabolites in responses to stress, damage, and infections caused by pathogens. Interestingly, these compounds are responsible for around 25% of drugs currently marketed, with examples included in cancer treatment [6,7,8,9]. Recently, the ability of some secondary plant metabolites to modulate estrogen signaling and hallmarks of cancer, such as proliferation and apoptosis, was discovered, which makes them applicable to the treatment of luminal BC, especially when resistant to endocrine therapy [10,11]. This review aims to summarize the characteristics of luminal BC, the mechanisms of resistance to endocrine therapy, and the potential of natural products to overcome this resistance through modulation of estrogen-receptor-related signaling.

## 2. The Role of Hormones in Mammary Gland Development

The female breast is characterized as an exocrine glandular structure, located in the anterosuperior wall of the thorax, and overlapping the pectoralis major muscle. It is composed of two large tissues: the stroma, consisting of adipocytes, fibroblasts, blood vessels, extracellular matrix, and inflammatory cells; and the epithelium, formed of branching ducts and lobes. This ductal–lobular system is covered by a layer of luminal cells, which are responsible for the secretory activity of the gland. Luminal cells are surrounded by basal myoepithelial cells that have the contractile capacity for lactation. Finally, this entire structure is still covered by a thin layer of epithelial tissue, in which the areola and mammary papilla are found [12,13,14,15].

**Figure 1:**
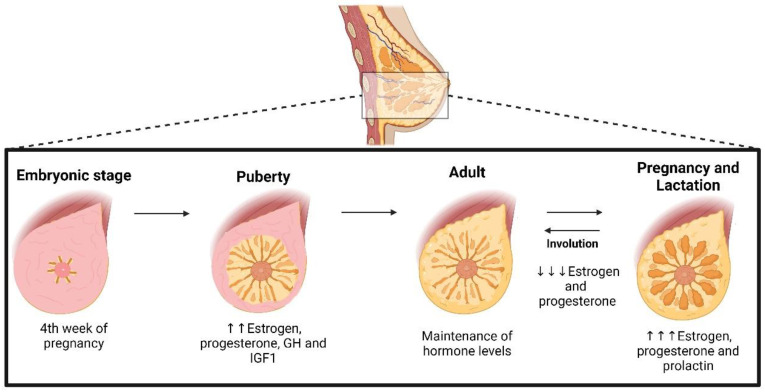
Representative scheme of the development process of the breasts, from the embryonic stage to pregnancy and lactation. In the embryonic stage, breast development begins, but the formation of ducts, alveoli, and fat deposition occurs mainly during puberty due to the increase in the expression and signaling of estrogen, progesterone, growth hormone (GH), and insulin-like growth factor (IGF-1). In adulthood, the development process continues in view of the stabilization of hormone levels. In pregnancy and lactation, the high levels of estrogen, progesterone, and prolactin increase the development of ducts and alveoli, promoting the production and release of milk. After this period, there is an involution of the breast. ↑ increased levels. Created with BioRender.com. Accessed on 23 September 2023.

Breast development occurs through different mechanisms and according to the stages of a woman’s life [16] (Figure 1). Hormonal stimuli allow the gradual evolution of the breast during the embryonic phase. In this stage, the embryonic ectoderm layer is responsible for forming the mammary lineage, which later organizes itself in regions of thickening, called placodes. These structures originate the rudimentary glandular ductal system. From birth until the onset of puberty, the mammary gland remains quiescent [13]. In the pubertal phase, there is an increase in the concentrations of progesterone and, mainly, of circulating estrogen, responsible for stimulating cell proliferation and breast growth, with greater fat gain and development of the ductal system. In addition, other hormones contribute to the formation of mammary ducts, such as growth hormone (GH) and insulin-like growth factor (IGF-1) [15,17].

However, it is only during pregnancy that the breasts actually reach maturity [13,18]. During pregnancy, estrogen, prolactin, and progesterone coordinate a set of changes in the mammary gland, stimulating cell proliferation and morphological changes in the alveoli, preparing them for the lactation process [12,17,19]. The mammary structure is mainly composed of adipose tissue, which decreases as the ductal system develops in response to increased levels of estrogen during pregnancy. Elevated concentrations of progesterone induce the development of the breast structure and lobular branching [12,20]. At the end of the process, estrogen and progesterone are found at high levels in the female organism, neutralizing the hormone prolactin and, consequently, inhibiting milk production. After childbirth, the decrease in estrogen and progesterone levels activates the lactation process [19]. With advancing age, hormone production tends to decrease and, when it stops, it triggers the onset of menopause [18].

The importance of hormones in the development of the mammary glands is evident. Initially, estrogen, an ovarian hormone, contributes significantly to the growth of the mammary glands during puberty [21]. Progesterone, in turn, acts in the alveologenesis of the gland, being responsible for the extensive development of lateral branches and alveoli—a process related to lactation [17,20,21]. Thus, all of the changes that occur in the breast are part of the natural cycle of the female organism, and the complete development of the mammary structure even protects against diseases such as BC. BC develops due to molecular dysregulation of ductal and lobular cells, and there are several risk factors for these tumors, including nulliparity and non-breastfeeding [22].

Epidemiological and experimental evidence has indicated estrogen levels to be an important risk factor for BC [16,23,24], including in postmenopausal women [25]. During menopause, estrogen production decreases considerably, causing uncomfortable symptoms. To control them, menopausal hormone therapies, also known as hormone replacement therapy, are adopted. However, these strategies have been associated with an increased risk of BC, particularly for ER-positive tumors [23]. Studies using animals also support the role of estrogen in the disease’s genesis and progression [26]. Therefore, it is evident that hormonal disorders play a key role in the pathogenesis of BC, so the modulation of estrogenic signaling has been presented as an important alternative in the treatment of the disease.

## 3. The Estrogenic Signaling

Normal breast and mammary stem cells’ development is regulated by different mechanisms involving ERs, HER2, and the Wnt/β-catenin signaling pathways, which control proliferation, cell death, cell differentiation, and motility [27]. Estrogen plays its physiological role through association with ERs, essentially as a cytoplasmic and nuclear signal that modulates the expression of different genes [28,29,30,31]

**Figure 2:**
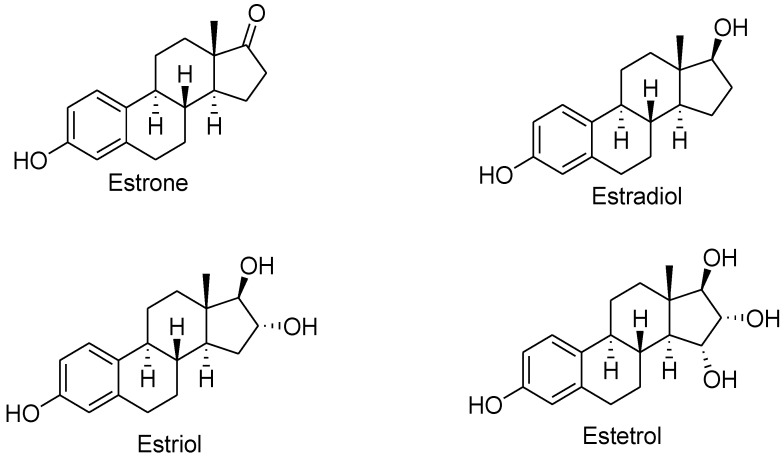
Chemical structures of the four main types of estrogens: estrone, estradiol, estriol, and estetrol.

Estrogens are steroid hormones structurally formed by four fused rings, three cyclohexenes, and one cyclopentane, with 17 carbon–carbon bonds. There are four main types: estrone, estradiol, estriol, and estetrol, all containing 18 carbon atoms (C_18_H_24_O_2_) and characterized by a benzene ring, a phenolic hydroxyl group, and a ketone group (in estrone) or one, two, or three hydroxyl groups (in different estrogens) [32,33] (Figure 2).

In mammalian females, estrogen synthesis occurs in the theca interna cells of the ovaries with the production of androgens, which are converted to estrogens in granulosa cells by the aromatase enzyme [34,35]. In this process, luteinizing hormone (LH), as found in Leydig cells, stimulates theca cells to synthesize androgens (such as androstenedione and testosterone) from cholesterol. On the other hand, the pituitary follicle-stimulating hormone (FSH) stimulates the granulosa cells of the ovarian follicles to express the aromatase enzyme, which is responsible for converting androgens into estrogens [36].

After synthesis, estrogen is released and passively penetrates cells due to its hydrophobicity. The most common form found in the circulation is 17β-estradiol (ETD) [37], which can also be produced in extragonadal cells, tissues, and organs, including the adrenal glands, mesenchymal cells, osteoblasts, chondrocytes, smooth muscle cells, endothelium, brain cells, adipose tissue, skin, and the pancreas [38]. In these other tissues, estrogen synthesis in postmenopausal women and men remains high, but it signals locally [33].

ERα, Erβ, and G-protein-coupled estrogen receptor 1 (GPER1) are directly involved in the effects of estrogens. ERα and ERβ belong to the subclass of nuclear hormone receptors and actively regulate gene expression [36]. ERα was the first to be discovered and has been extensively studied. ERβ, with significant structural similarities to ERα, was identified almost four decades later, showing distinct and non-redundant roles. Subsequently, the association of GPER1 with cytoplasmic estrogen signaling was established [33,36,39].

ERα and ERβ are encoded by different genes (*ESR1* and *ESR2*, respectively), have differences in their DNA-binding domains and estrogen-binding domains, and can activate different genes. Although these receptors can form homodimers and heterodimers, they show distinct patterns of expression in mammary cells, as well as different physiological and pathological effects [33,36,39]. GPER1, in turn, is a membrane receptor that does not directly change gene expression, since it does not have recognition sites in chromatin. Therefore, it activates second messengers, which can alter the cellular metabolic profile and, in a secondary and late way, the expression of some genes [40].

**Figure 3:**
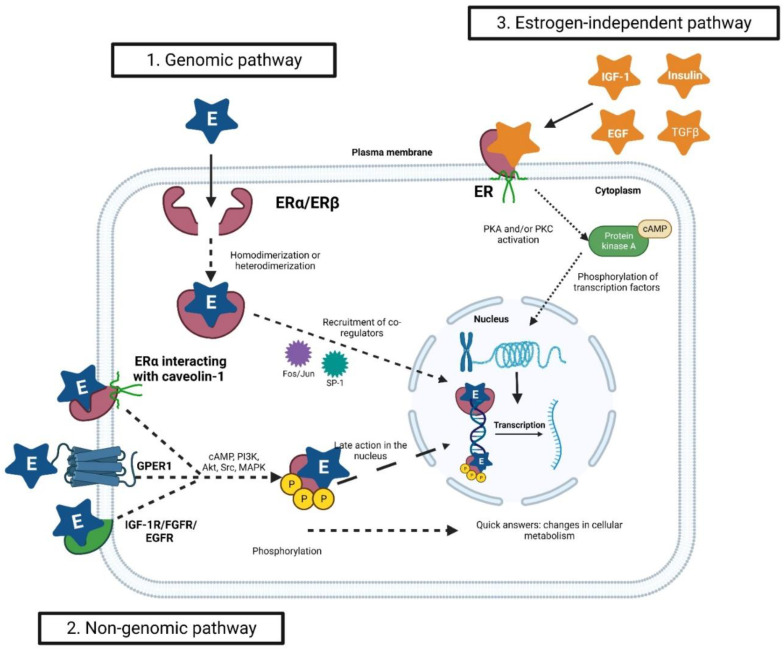
Representative scheme of estrogen receptor (ER) signaling pathways: (1) Genomic pathway: after binding to estrogen (E), the ER dimerizes (homodimerizes or heterodimerizes), translocates to the nucleus, and exerts its regulatory functions. (2) Non-genomic pathway: the ER drives signaling through the plasma membrane, activating cytoplasmic signal transduction mechanisms. (3) Estrogen-independent pathway: the ER can be modulated by extracellular signals without estrogen. P (phosphate), cAMP (cyclic adenosine monophosphate), PI3K (phosphatidylinositol 3-kinase), Akt (protein kinase B), MAPK (mitogen-activated protein kinase), IGF-1 (insulin-like growth factor), EGF (epidermal growth factor), ERE (estrogen-response element). Created with BioRender.com. Accessed on 23 September 2023.

The action of estrogens involving ERs in target cells can occur through three different pathways, isolated or together: (i) the genomic pathway, (ii) the non-genomic pathway, and (iii) the estrogen-independent pathway [41,42] (Figure 3). In general, in the genomic pathway, estrogen activates ERs in the cell’s cytoplasm, which leads to dimerization (homodimerization or heterodimerization), translocation to the nucleus, and regulation of gene expression. In the non-genomic pathway, ERs drive signaling across the plasma membrane, activating cytoplasmic signal transduction mechanisms. In the estrogen-independent pathway, ERs can be activated by molecules other than estrogen [32,33].

### 3.1. The Genomic Pathway

In the genomic pathway, the free steroid enters the target cell through passive diffusion across the plasma membrane and then binds to the ER with high affinity. Once estrogen–ER binding is established in the cytoplasm, the receptor is phosphorylated, changes its conformation, and dimerizes. This complex then interacts with co-regulatory proteins and is translocated to the nucleus, where it binds to chromatin in specific sequences called estrogen-response elements (EREs) [43,44]. These interactions promote the transcription of genes that act in the regulation of different cellular processes, including the cell cycle, DNA replication, cell differentiation, and apoptosis [32,45,46,47].

The activated ER is capable of binding to over 10,000 sites throughout the genome. Furthermore, this pathway can indirectly activate the expression of genes that lack EREs, through the recruitment of different co-regulators. Co-regulators can promote post-translational histone modifications, interact with transcription factors (e.g., Fos/jun; SP-1), or even directly regulate the binding or activity of RNA polymerase II. Thus, the ER alters the transcriptome of hormone-responsive cells [30,48,49]. Studies have shown that the distinct combination of co-regulatory elements with the ER may be one of the main factors responsible for the clinical course of tumors [50,51,52,53]. In fact, in addition to activating genes related to the cell cycle of normal cells, estrogens, through the genomic pathway, can activate genes with oncogenic potential, such as *MYC* proto-oncogene, *CCND1* (cyclin D1), *FOXM1* (forkhead box M1), *GREB1* (growth-regulating estrogen-receptor-binding 1), *BCL2* (B-cell leukemia/lymphoma 2 apoptosis regulator), amphiregulin, *IGF1*, and *CXCL12* (C-X-C motif chemokine ligand 12) [54].

### 3.2. The Non-Genomic Pathway

In the non-genomic estrogen mechanism, however, cellular responses are fast, suggesting the occurrence of short-term cytoplasmic events, in addition to late action in the nucleus. Indeed, while genomic effects occur on the timescale of hours, some steroid-induced nuclear events can occur within minutes [43]. The non-genomic pathway relies on steroid receptors in the plasma membrane and activates signal transduction mechanisms, with the subsequent production of intracellular second messengers, indirectly changing gene expression [32,55]. A small group of ERα located in the extracellular compartment or close to the membrane is involved in membrane-initiated non-genomic steroid signaling. This receptor location is, in part, due to its direct interaction with caveolin-1 in response to post-translational modifications such as palmitoylation [56]. Moreover, GPER1, independent of ER, binds to estrogen and activates the mitogen-activated protein kinase (MAPK) and epidermal growth factor receptor (EGFR) pathways. Evidence confirms that ETD binds directly to GPER1 [57]. In addition, insulin-like growth factor 1 receptor (IGF-1R), fibroblast growth factor receptor (FGFR), and EGFR can recognize estrogens and activate the phosphatidylinositol 3-kinase (PI3K)/protein kinase B (Akt) and MAPK pathways [43,58,59].

### 3.3. The Estrogen-Independent Pathway

Finally, ERs can be activated independent of ETD or other specific ligands. Different molecules can act as ligands, including insulin, IGF-1, TGFβ, and epidermal growth factor (EGF). Regulators of cellular phosphorylation, such as protein kinase A (PKA) or protein kinase C (PKC), as well as extracellular signals such as growth peptides, cytokines, neurotransmitters, and cell-cycle regulators, are also involved. These findings expand our understanding of the complexity of ER signaling pathways beyond traditional estrogen-induced genomic and non-genomic responses [33,56,60,61,62].

A crosstalk of different mechanisms is related to the occurrence and progression of malignant cells, and estrogen and its receptors (ERα, Erβ, and GPER1) are directly associated with BC. In these tumors, estrogen regulates the cell cycle and metabolism, responding to the high energy demand of tumor cells [50]. Furthermore, the correlation between estrogen metabolism and increased production of reactive oxygen species (ROS) has already been described [63].

ERα has a widely described role in BC, promoting disease progression. ERβ, in turn, has shown a controversial effect, and studies are still needed to elucidate its role in BC. ERβ expression is reduced by about 80% in tumors, and its activation inhibits cell replication, stimulates apoptosis, and increases the sensitivity of these cells to chemotherapy treatments. However, there are reports of a pro-tumorigenic action of ERβ [64,65,66,67]. GPER1 also presents a contradictory action in BC. Its low expression has already been correlated with lower overall survival [67]. However, in patients with ER-positive BC, GPER1 was correlated with hormone therapy resistance and aggressive disease recurrence [68]. Therefore, a molecular understanding of BC is essential, especially for the development of effective strategies that promote a better quality of life for patients.

## 4. Molecular Subtypes of Breast Cancer

Histologically, invasive ductal BC is the most commonly diagnosed subtype (50–75% of patients), followed by invasive lobular carcinoma (5–15% of patients) [69]. The histopathological classification has diagnostic and prognostic value. However, alone, it does not cover the heterogeneity of BC, limiting greater accuracy in the evaluation of the clinical course of the disease, and calling for an assertive decision about the treatments to be adopted [70]. In this context, the analysis of the BC’s molecular profile based on the expression of ERα, PR, and HER2, as first established in 2000 by Perou et al. [71], is essential for patient management, and breast tumors are currently grouped into four main subtypes: (i) luminal A, (ii) luminal B, (iii) HER2-enriched (HER2E), and (iv) triple-negative (TN) [72].

The luminal subtypes are tumors that usually begin in the epithelial cells that surround the lumen of the duct [73]. Luminal A is the most common, representing about 50–60% of diagnosed BCs [70,74]. It is positive for ERα, with PR levels greater than 20%, negative for HER2, and with a percentage of Ki67 (a proliferation index marker) lower than 14%, being associated with a lower risk of recurrence and a better prognosis [75].

Luminal B tumors represent about 10 to 20% of diagnosed BCs [76]. They have a higher proliferative profile than luminal A tumors and overexpress growth receptor signaling genes, with approximately 20% of luminal B tumors being HER2-positive [77]. In this scenario, there is a differentiation between HER2-negative and HER2-positive luminal B tumors. HER2-negatives are generally ERα-positive and express low levels of PR. However, Ki67 expression is higher than 14%. HER2-positive luminal B tumors, in turn, express Erα and have a variable status for Ki67 and PR [78], but they overexpress HER2, a member of the family of four membrane tyrosine kinases, whose heterodimerization activates a signaling cascade that promotes proliferation, survival, and metastasis [79,80]. They respond to anti-HER2 monoclonal antibodies, including trastuzumab, pertuzumab, and trastuzumab emtansine conjugation (T-DM1), as well as inhibitors of receptor tyrosine kinase activity (e.g., lapatinib and neratinib) [81,82]. Luminal B BC tends to be diagnosed in younger women compared to luminal A BC [80,83]. Bone recurrence is frequent and can reach other organs, with lower survival rates compared to patients diagnosed with the luminal A subtype [83,84]. It is worth noting that luminal A and B tumors both have expression patterns associated with the luminal mammary epithelium, such as ERα and luminal cytokeratins (CKs) (CK8 and CK18) [85,86]. In fact, ERα is expressed in 70–75% of patients with invasive carcinomas, and PR is expressed by approximately 50% of ERα-positive patients but rarely expressed in patients with ERα-negative BC [87]. These findings suggest the regulation of PR expression by ERs, and a lower expression of PR is associated with a more aggressive disease [88,89].

In HER2E tumors, in about 15 to 20% of cases, there is an overexpression of HER2, being negative for hormone receptors. Thus, they present highly proliferative cells, and 75% of these tumors have a high histological grade, while 40% present mutations in the *TP53* gene [90,91]. Despite being eligible for anti-HER2 therapies, bone metastases are recurrent, the rate of visceral recurrence is high, and a shorter disease-free survival has been reported [92,93].

TNBC does not express ERα, PR, or HER2. It accounts for about 15–20% of invasive BCs, being more common in women under the age of 40 years, African-American women, and patients with *BRCA1/BRCA2* mutations [94,95]. Histologically, TNBC is poorly differentiated, highly proliferative, and presents heterogeneous tumors with subtypes of variable prognosis, namely, basal-like 1 (BL1), basal-like 2 (BL2), mesenchymal (M), mesenchymal stem-like (MSL), immunomodulatory (IM), and luminal androgen receptor (LAR) [96]. In immunohistochemical analyses, they are subdivided into basal and non-basal tumors, with the basal ones expressing CK5/CK6 and EGFR1 [97,98]. TNBC is associated with a worse prognosis, early recurrence, high frequency of metastases in the lungs, liver, and brain, and lower survival [95,99].

Another important factor for the molecular understanding of BC subtypes is the expression of ERβ, which has been shown to be more common in luminal BC than in the TN or HER2E subtypes [64]. Recently, RNA-Seq assays using BC samples indicated that *ESR2* is less expressed than *ESR1*, with *ESR2* being associated with greater overall survival and modulation of the immune response [100]. Thus, new molecular subtypes have been identified. In fact, molecular classification has reshaped the diagnosis of BC and enabled the identification of new targets, substantially contributing to the development of new therapeutic approaches.

## 5. Treatment for Luminal Tumors

In addition to the molecular subtype, tumor staging, age, and comorbidities guide therapeutic decisions for BC [101,102]. Surgery is adopted according to the size of the tumor, along with its stage, histological classification, and presence of metastases [103]. Currently, strategies include complete surgical removal of the breast (mastectomy) and conservation surgery (quadrantectomy), followed or not by radiotherapy [104]. In recent years, most studies on the recurrence and post-surgical metastasis of BC have focused on non-luminal cases, due to their worse prognosis. However, luminal tumors, despite often being diagnosed early (mainly through mammographic screening and surgery), have a high recurrence rate [105].

Radiotherapy and chemotherapy are commonly used in the treatment of BC, being performed either alone or as adjuvant therapy [106]. Radiotherapy is based on ionizing radiation directed to the affected tissue, which is efficient in decreasing the tumor size. However, depending on the tumor extension, different amounts of sessions and radiation are indicated and, consequently, different side effects are observed. Because it is a local treatment, radiotherapy is often not able to eliminate circulating tumor cells [106,107]. Luminal A BC receives the greatest benefit in this treatment modality, while HER2E and TN tumors are less responsive. It is believed that the degree of invasiveness, malignancy, and radiosensitivity of these tumor subtypes affect the effectiveness of the treatment [108].

Chemotherapy, in turn, consists of using chemical compounds systemically, targeting normal and malignant cells. Thus, it is responsible for debilitating side effects. The development of intrinsic or acquired resistance by tumor cells is also common [106,109,110,111]. In luminal BC, the effects of adjuvant chemotherapy have been questioned [112]. Evidence indicates that luminal A tumors are not sensitive to chemotherapy with paclitaxel and doxorubicin, compared to more aggressive tumors [113,114]. However, current international guidelines recommend the use of anthracyclines/taxanes as the standard cytotoxic regimen for early-stage BC that does not express HER2 [115,116].

ERα-positive tumors are eligible for hormone therapy (endocrine therapy), with an average duration of 5 to 10 years [117]. Endocrine therapy aims to slow down or stop the growth of estrogen-dependent tumors, either by blocking hormonal effects on transformed cells or preventing their synthesis in the body [118]. Currently, endocrine therapy for BC consists of (i) selective ER modulators (SERMs), such as tamoxifen; (ii) selective ER downregulators (SERDs), such as fulvestrant; (iii) aromatase inhibitors (AIs), such as letrozole, anastrozole, and exemestane; and (iv) ovarian function suppressors, combined or not with chemotherapy [78,119].

SERMs compete with estrogen for binding to ERs, with agonist or antagonist characteristics, depending on the target tissue. On the other hand, SERDs allow for the formation of an unstable protein complex that induces ERα degradation [120]. For young women, SERMs are indicated [117,121], and because they regulate the receptor, they are also prescribed for postmenopausal women [106,122]. Interestingly, for women at greater risk of developing BC, long-term hormone therapy can be used to prevent the disease, reducing the probability of occurrence by up to 50% [123,124].

Tamoxifen is the most widely used SERM and selectively blocks signaling at the ERα level, inhibiting cell proliferation [125,126]. Tamoxifen is a prodrug that is metabolized in the human liver, predominantly by the cytochrome P450 (CYP) system, into primary and secondary metabolites [127]. Endoxifene and 4HT are the main active metabolites, binding to the receptor with similar affinity and potent cytotoxic action [128,129]. The benefit of using tamoxifen to treat women with ERα-positive BC is widely described. It is known that the administration of tamoxifen for 10 years significantly reduces the risk of recurrence, in addition to promoting a significant increase in the overall survival of patients, compared to treatment for only 5 years [130]. It should also be noted that other SERMs analogous to tamoxifen have been proposed, such as toremifene and raloxifene, both of which have been approved by the US Food and Drug Administration (FDA), with the aim of increasing the efficiency of hormone therapy and limiting side effects [131]. ERβ has also been investigated as a therapeutic target in BC, but its clinical application is limited by the lack of selective agonists [132].

SERDs block receptor activity by promoting its degradation via the proteasome and, therefore, have anti-estrogenic effects [133]. Fulvestrant, the only FDA-approved SERD for the treatment of BC, has about 100-fold greater affinity for ERα compared to tamoxifen, with no side effects on uterine tissue [134,135]. It is used in patients with advanced breast tumors and as a second-line therapy for those resistant to tamoxifen [136]. Furthermore, fulvestrant may sensitize ERα-negative breast tumor cells to chemotherapy, showing a synergistic action with cytotoxic agents such as docetaxel [137]. However, this drug has shown low bioavailability and a controversial neoadjuvant effect [135,138].

AIs decrease estrogen production from androgens [139,140]. Pre-menopause, estrogen production occurs mainly in the ovaries, being significantly reduced in advanced age. Post-menopause, estrogen available to the body is produced in smaller amounts in adipose tissue and depends on aromatase activity [122], which justifies the effectiveness of drugs specifically targeted at these enzymes. As aromatase has high specificity and is involved only in the last step of estrogen biosynthesis from testosterones, its inhibition does not affect the levels of other biologically important steroids [141].

Exemestane is a steroidal AI that irreversibly inhibits aromatase by acting as a false substrate for the enzyme, thus suppressing estrogen biosynthesis, mainly in peripheral adipose tissues [142]. Exemestane therapy has been shown to be effective in reducing BC recurrence and mortality rates compared to tamoxifen [143]. Anastrozole and letrozole, in turn, are non-steroidal AIs that reversibly inhibit the enzyme, being administered for up to five years after the end of adjuvant chemotherapy [131,144,145]. Their benefits over tamoxifen are evident, such as increased disease-free survival, especially in patients with advanced stages of BC [143,146]. For the use of AIs in premenopausal women, ovarian suppression should be performed, with the administration of gonadotropin-releasing hormone (GnRH) agonists [78]. In addition, the use of GnRH with SERMs has increased disease-free and overall survival [147].

The use of SERMs, SERDs, and AIs in the treatment of BC is associated with side effects related to estrogen deprivation [148]. Patients report hot flashes, weight gain, sexual dysfunction, osteoporosis, and musculoskeletal symptoms, which can compromise treatment [149,150]. Tamoxifen has been associated with more serious side effects compared to other agents, while exemestane causes musculoskeletal symptoms and hot flashes [151]. Patients treated with AIs report gastrointestinal symptoms such as nausea, vomiting, and diarrhea, while the incidence of thromboembolic events and vaginal bleeding is lower compared to the administration of SERMs and SERDs [148,152]. Finally, it is believed that approximately 30 to 40% of ERα-positive BC are resistant to endocrine therapy, which leads to a higher rate of recurrence and a worse disease prognosis [153,154]. New SERMs and SERDs are under development, capable of reducing ERα expression or activity and blocking estrogen-dependent and estrogen-independent ERα signaling. These inhibitors are therefore considered to be a significant and promising therapeutic approach to treat luminal tumors, both in early stages and in more advanced cases, especially when resistant to traditional strategies [155,156].

## 6. Resistance to Hormone Therapy

**Figure 4:**
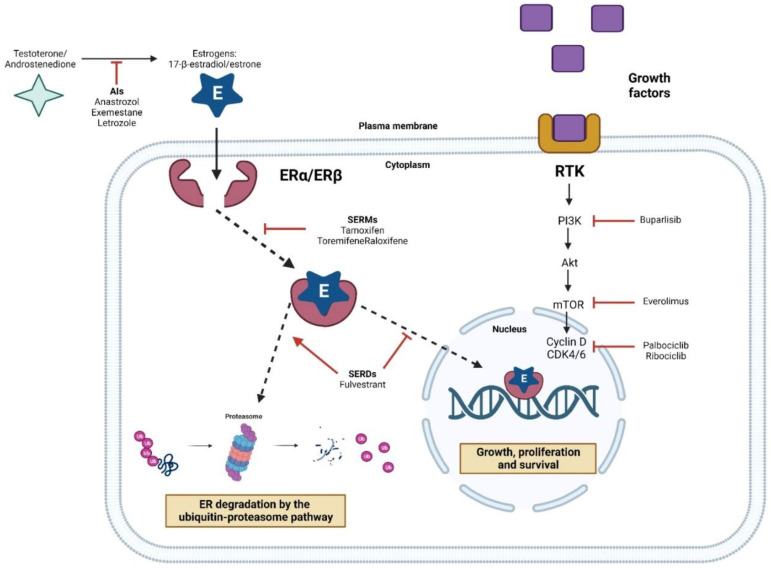
Mechanism of action of hormone therapy and other active compounds in breast cancer: E (estrogen), ERα/ERβ (estrogen receptors α and β), RTK (receptor tyrosine kinase), PI3K (phosphatidylinositol 3-kinase), Akt (protein kinase B), mTOR (mammalian target of rapamycin), CDK4/6 (cyclin-dependent kinases 4 and 6), SERMs (selective ER modulators), SERDs (selective ER downregulators), AIs (aromatase inhibitors). Created with BioRender.com. Accessed on 23 September 2023.

Although endocrine therapy is essential for the treatment of luminal tumors, with clinically significant benefits in disease-free survival and overall survival, the efficacy of hormone therapy is still limited in the face of de novo (primary) or acquired (secondary) tumor resistance. De novo resistance develops early on or over the course of treatment, usually within the first two years. Acquired resistance occurs due to unresponsiveness and tumor growth after the end of endocrine therapy [126,157]. Of all ERα-positive tumors, only 50% are responsive to the first administration of antiestrogens, e.g., tamoxifen. Furthermore, metastatic tumors, although initially responsive, end up becoming resistant to endocrine therapy, which substantially worsens the patient’s clinical condition, leading to death [158,159,160]. Endocrine resistance mechanisms mainly include the dysregulation of ERα expression, mutations and epigenetic changes recruiting different co-activators/co-repressors, expression of ERβ and ERα isoforms, and increased activity of receptor tyrosine kinases (RTKs) (Figure 4) [161].

As ERα activation is essential for tumor cells’ proliferation and differentiation, loss of receptor expression in BC is one of the main causes of resistance to endocrine treatment [162,163]. In fact, ERα expression may change during disease progression, and conversion of ERα-positive to ERα-negative tumors may occur in approximately 10–20% of patients [164]. Additionally, mutations in *ESR1* are described in 20–40% of metastatic cases previously treated with hormone therapy [165,166]. Most of these mutations affect the estrogen-binding domain on the receptor and promote constitutive ERα activity [167]. Moreover, epigenetic modifications are also critical regulators of physiological function. In this scenario, phosphorylation, acetylation, methylation, and ubiquitination can alter receptor stability, subcellular localization, transcriptional activity, and DNA-binding capacity [168]. ERα phosphorylation at Ser104/106 and Ser305, for example, contributes to tamoxifen resistance [169,170]. Furthermore, mutations in *ESR1* on Ser305 may also confer resistance to AIs [171]. DNA methylation and histone acetylation are also described as epigenetic changes that are capable of promoting resistance to endocrine therapy. For example, *ESR1* hypermethylation reduces ERα expression by 20% in patients treated with tamoxifen [172]. These alterations (mutational and epigenetic) promote the recruitment of different co-activators and/or co-repressors that subsequently activate other oncogenic signaling pathways such as those related to HER2, FGFR, and IGF-1R, directly affecting the response to hormone therapy [161,165,173,174].

Evidence suggests that ERβ expression levels may also be an important predictor of the response to tamoxifen [175]. Higher ERβ expression is more frequent in tamoxifen-responsive patients compared to those who are resistant to endocrine therapy [176,177]. In addition, phosphorylation and nuclear localization of ERβ are associated with a better disease prognosis, even in cases that are resistant to tamoxifen treatment [178]. ERβ can be inactivated by its antagonist ERα-36, an isoform of ERα. Overexpression of ERα-36 is observed in TNBC and luminal tumors that are resistant to endocrine therapy, with reduced ERα expression. Thus, lower expression of ERα in luminal BCs may be associated with increased expression of ERα-36 [179,180].

RTKs are a family of receptors attached to the cell membrane, whose intracellular domain contains a tyrosine kinase capable of autophosphorylation or phosphorylation of tyrosine residues in target proteins [181,182]. Examples of RTKs include EGFR, IGF-1R, and vascular endothelial growth factor receptors (VEGFRs). These are activated after interaction with the ligand, including growth factors, cytokines, or hormones [183,184,185]. Upon interaction with the ligand, intracellular signal transduction pathways are initiated, such as MAPK and PI3K/Akt, which are associated with endocrine resistance. In BC, these pathways activate ERα transcriptional activity in the absence of estrogen signaling. Furthermore, RTKs may decrease ERα expression, since the signaling is ER-independent [186,187].

Somatic mutations in genes encoding PI3K/Akt regulators occur in up to 70% of BCs, with the most frequent being those observed in the PI3K catalytic subunits, as well as in PI3K modulators such as PTEN, Akt, and mTOR [188]. These mutations promote hyperactivation of PI3K kinase activity, worsening the prognosis of late-stage luminal breast tumors [189]. Another important downstream effector of the PI3K/Akt pathway is the mTOR complex. This is composed of two interdependent factors: mTORC1 and mTORC2, whose increased kinase activity promotes BC growth and proliferation [190]. The tumor suppressor PTEN is a negative regulator of mTOR, and it is known that BC patients with germline mutations in *PTEN* are at increased risk of developing a second breast tumor, as well as endometrial, thyroid, renal, and colorectal cancers [191].

Cyclin D1 and cyclin-dependent kinase (CDK) signaling have also been associated to resistance. In vitro assays demonstrated that luminal BC cells in which cyclin D1 expression was induced continued to proliferate even with the administration of tamoxifen [192,193]. Furthermore, higher levels of *CCND1* transcripts were identified in patients with luminal BC and correlated with a shorter disease-free survival time and shorter overall survival [192,194,195,196]. Therefore, as a complement to hormone therapy, different chemotherapy agents have been evaluated, including CDK 4/6 inhibitors (e.g., palbociclib, ribociclib), epigenetic modulators that inhibit histone deacetylase (HDAC), and mTOR inhibitors [197,198]. Experimentally, the combination of fulvestrant with CDK4/6 inhibitors was evaluated in patients who were resistant to conventional endocrine therapies. In addition, some benefits have also been observed with the use of HDAC inhibitors in these treatments [199,200]. The mTOR inhibitor everolimus, combined with exemestane, has also improved progression-free survival in patients with AI-resistant advanced luminal BC [201].

Thus, the identification and characterization of new active compounds for luminal BC is essential for the development of innovative and assertive therapeutic strategies, especially those capable of overcoming endocrine resistance. In this context, with the focus on new effective treatments for BC with fewer side effects, phytochemicals have emerged, offering structural and functional versatility. In fact, the susceptibility of ERs to herbal medicines has been recognized [202,203].

## 7. Natural Compounds and Their Effects on Luminal Tumors

Plants are considered to be important sources of substances for the treatment of cancer, being effective, safe, and with structures subject to modification. Natural products have antioxidant, growth-inhibiting, apoptosis-inducing, and invasion- and metastasis-control activities [204,205,206]. Despite advances in scientific studies focused on this area, it is estimated that only 15% of existing plant species have already been investigated for their pharmacological potential [207]. Therefore, the need to develop effective natural therapeutic agents is recognized, especially in the face of therapeutic resistance.

Natural products have been used in adjuvant therapy and proven to be versatile and capable of modulating hormonal signaling, interfering with the cell cycle, proliferation, invasion, metastasis, and angiogenesis [208]. Phytoestrogens, for example, are natural compounds derived from plants and are analogous to estrogens in structure and function [11,209]. In addition, other natural compounds have been shown to be active in luminal tumors, capable of reversing cases of resistance to hormone therapy [210,211,212]. The richness of the therapeutic potential of plants is due to the presence of active phytochemicals, and herein we present information about natural compounds that have been explored for the control of luminal BC. Table 1 summarizes the main natural products, their classes, and their effects on luminal BC cell lineages.

### 7.1. Flavonoids

**Figure 5:**
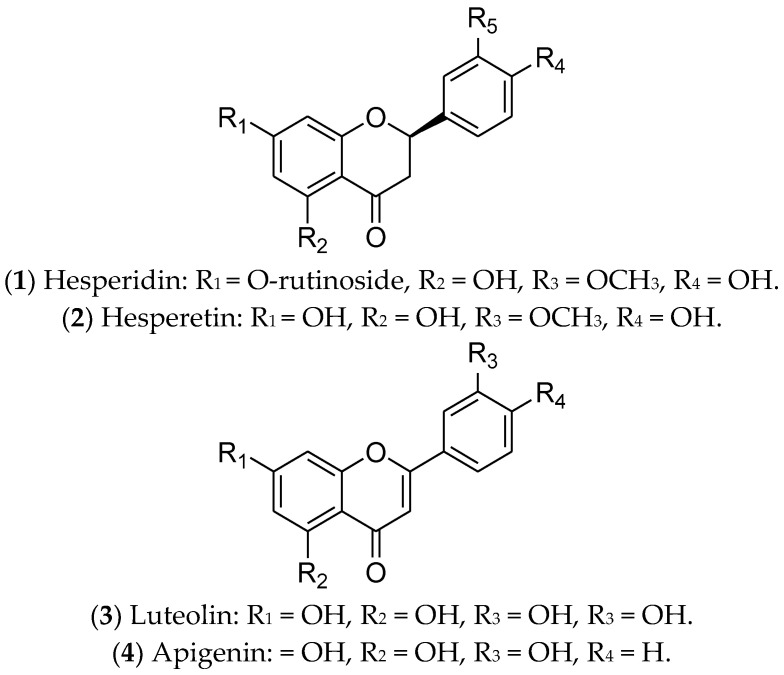
Flavonoids with effects on luminal breast cancer.

Flavonoids are polyphenolic secondary metabolites that have been evaluated for the treatment of BC due to their antitumor potential through epigenetic changes, expression of tumor-suppressor genes, and activation of pro-apoptotic pathways. Interestingly, flavonoids can modulate ERs, especially hesperidin, hesperetin, luteolin, and apigenin [251,252,253] (Figure 5).

Hesperidin (**1**) is a glycosylated flavanone compound composed of hesperetin (**2**) (main part) and a disaccharide called rutinose (a type of glucose-linked rhamnose) [213]. Hesperidin and its derivatives are found in citrus fruits from the Rutaceae family, such as oranges, tangerines, limes, lemons, and grapefruit [254]. They have antimitotic, pro-apoptotic, antimetabolic, and antimetastatic potential, due to their anti-inflammatory and antioxidant properties [213,253]. These compounds, separately or in combination, have been used in the treatment of luminal BC cells (MCF 7 and T-47D) and have been responsible for downregulation of *ER1* expression. Therefore, hesperidin and its derivatives can modulate estrogenic signaling [213,255,256]. In vivo assays using an MCF 7 xenograft model demonstrated that hesperidin inhibits tumor growth and metastasis, mainly through overexpressing estrogen synthase-aromatase [257]. To date, there are no reports of clinical trials with these compounds. Despite their relevant biological functions, both hesperidin and hesperetin have poor water solubility and limited bioavailability. For this reason, several studies have been focused on creating nanoformulations to increase their bioavailability [257].

Luteolin (**3**) is a natural flavonoid that regulates cancer-related signaling pathways. It is commonly found in carrots, broccoli, sweet peppers, celery, parsley, onion leaves, and chrysanthemum flowers [216]. Recent evidence has shown that luteolin promotes cell death by apoptosis, acting as an antioxidant and anticancer agent in different tumors, including BC [215,216]. In luminal BC cells, luteolin upregulates caspases 3, 8, and 9, as well as BAX (Bcl-2-associated X-protein—pro-apoptotic regulator) and miR-16. Also, this compound downregulates the expression of BCL-2 (an anti-apoptotic protein) and inhibits IGF-1 activation by modulating the P13K-Akt signal transduction pathway [258]. Furthermore, the combination of luteolin with Taxol is synergistic and can increase the sensitivity of BC cells to the treatments adopted [259]. An interesting study conducted by Markaverich and collaborators demonstrated that the treatment of MCF 7 cells with luteolin modulated different genes of the estrogen pathway, such as *GTF2H2* (general transcription factor IIH, polypeptide 2) (-), *NCOR1* (nuclear receptor co-repressor 1) (-), *TAF9* (+), *NRAS* (neuroblastoma viral RAS (v-ras) oncogene homolog) (-), *NRIP1* (nuclear receptor interacting protein 1) (-), *POLR2A* (polymerase (RNA) II (DNA-directed) polypeptide A) (-), *DDX5* (DEAD (Asp-Glu-Ala-Asp) box polypeptide 5) (-), and *NCOA3* (nuclear receptor co-activator 3) (-) [260]. The joint action of luteolin and indole-3-carbinol effectively inhibited ER-positive BC. This treatment targeted two key therapeutic elements—ERα and the CDK 4/6/retinoblastoma (Rb) pathway—in cell lines and xenograft tumors [261]. Currently, there are no ongoing or completed clinical trials using luteolin in the treatment of BC patients.

Finally, apigenin (**4**) is a flavonoid that is found in species belonging to the Asteraceae family [262]. This compound inhibits cell growth and induces apoptosis of luminal BC cells through the regulation of caspases, cytochrome c release, the NF-κB, PI3K, and Akt/mTOR pathways [263,264], and poly-ADP ribose polymerase (PARP) cleavage [217,218]. In vivo, Yao and colleagues demonstrated that apigenin partially antagonizes ERs [265]. However, this compound is poorly soluble in water, and nanotechnology has contributed to advances related to its therapeutic applications [266]. Although promising for the treatment of luminal BC, there are no reports of completed or ongoing clinical trials.

### 7.2. Isoflavonoids

**Figure 6:**
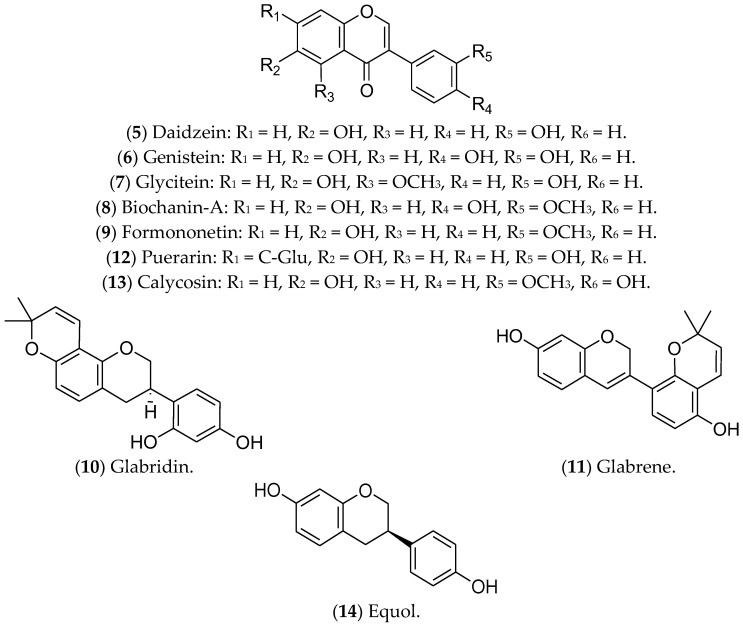
Isoflavonoids with effects on luminal breast cancer.

Isoflavonoids, or isoflavones, are phytoestrogens that have demonstrated greater affinity for ERβ [267,268]. The ingestion of isoflavones either in childhood or in puberty seems to contribute to the prevention of BC [268]. Regarding treatment of luminal BC, the isoflavones daidzein, genistein, glycitein, biochanin A, formononetin, glabridin, glabrene, puerarin, calycosin, and equol have already shown promising effects (Figure 6).

Daidzein (**5**) is an isoflavone present in soybeans that shares similarities with human estrogens, acting in a dual way: replacing or blocking the action of the hormones in ERs [269,270,271]. In luminal BC cells, such as MCF 7 cells, daidzein inhibits the NF-kB pathway, CYP1, and topoisomerase, leading to cell-cycle arrest and apoptosis [272]. A phase I multiple-dose clinical investigation indicated that this compound was safe in healthy postmenopausal women (ClinicalTrials.gov Identifier: NCT00491595) [273].

The isoflavone genistein (**6**) is a phytoestrogen found in soy and its derivatives [274] that inhibits ER-positive BC tumors, suppressing MAPK and DNA polymerase II, reducing cell proliferation, and triggering apoptosis [270,275]. This compound also increases ERβ and decreases ERα expression at the transcriptional and protein levels [276,277]. Genistein has been recognized as one of the most biologically active and potent isoflavones for cancer prevention [270,275]. However, it is noteworthy that studies in animal models found that genistein (**6**) and daidzein (**5**), even at lower concentrations, promote the development of BC, highlighting the need for further experiments focused on the characterization of its metabolites and their effects on breast tumors. Moreover, in in vitro tests, these substances counteracted the antitumor effects of tamoxifen [278,279].

The compounds glycitein (**7**), biochanin A (**8**), formononetin (**9**), glabridin (**10**)**,** and glabrene (**11**) are phytoestrogens that are capable of positively regulating BCL-2 and modulating important oncogenic pathways such as IGF-1/IGF-1R, MAPK, and P13K/Akt [227]. Glycitein (**7**), an O-methylated isoflavone that is present in soybean foods, can decrease the glucose uptake of MCF 7 cells and modulate the metabolic status of ERα-positive cells [225]. The results available in the literature about glycitein are limited to in vitro assays. Biochanin A (BCA) (**8**) is an isoflavonoid that is present in large quantities in chickpeas, soybeans, red clover, and other herbs [280]. BCA can reduce migration/invasion and activate pro-inflammatory pathways of MCF 7 cells through ROS production and inhibition of the ERK-1/2 pathway [281,282]. In addition, BCA has a preventive effect against BC, whether administered alone or combined with other flavonoids [283]. In vivo assays demonstrated that BCA has a synergistic effect with 5-fluorouracil, reducing tumor size, mainly associated with the ER-α/Akt axis [284].

Formononetin (**9**), in turn, is an active component extracted from the traditional Chinese medicinal herb *Astragalus membranaceus* that can reverse resistance to chemotherapy. This compound regulates the expression of *CXCL12*, *ESR1*, and *IGF1*, modulates the Akt and mTOR pathways, and increases the sensitivity of Taxol-resistant BC cells [285,286]. In nude xenograft mice, the treatment with formononetin controlled the growth of BC [287]. Preclinical assays have also demonstrated the ability of this compound to inhibit angiogenesis through modulating the Akt pathway and downregulating the effect of basic fibroblast growth factor 2 (FGF2). There are no ongoing clinical trials [288,289].

Glabridin (**10**), a flavonoid from the root of *Glycyrrhiza glabra,* traditionally called licorice, has antiproliferative activity in MCF 7 cells [290], associated with oxidative stress, mitochondrial dysfunction [291], and modulation of EGFR expression [292]. Similar to glycitein (**7**), this flavonoid modulates ERs, with a proliferative effect at lower concentrations and an antiproliferative effect at higher concentrations [293]. In an animal model of TNBC, glabridin combined with a low concentration of paclitaxel significantly reduced the tumor burden and the formation of lung metastases [294]. Glabrene (**11**), also present in licorice, has a higher affinity for ERs and a dual effect on BC cells; at lower concentrations (10 nM–10 µM), it promotes ER-dependent growth, while at higher concentrations (>15 µM) it shows ER-independent antiproliferative activity [293]. In vivo studies have demonstrated that glabridin is similar to ETD, but both glabridin and glabrene have limitations for the treatment of luminal BC, especially given the lack of validation of their therapeutic effects [295].

Puerarin (**12**), a natural isoflavone from *Pueraria lobata* (a plant from China and Japan known as the “kudzu vine”), has therapeutic potential for luminal BC cells, inhibiting cell migration, adhesion, and invasion, and triggering apoptosis through modulation of non-coding RNAs (ncRNAs) [296]. Furthermore, treatment with puerarin reduces multidrug resistance in Adriamycin-resistant MCF 7 cells [297]. In particular, the action of puerarin in the estrogenic pathway in luminal BC has not yet been described, but in vivo studies indicate that this compound increases the expression of ER-α in cardiac tissues in ovariectomized animals [298]. A previous study investigating the anti-osteoporotic action of puerarin showed that this compound weakly binds to ERs [299]. However, as the results with puerarin in BC are limited to a few in vitro experiments, its clinical application is still incipient.

Calycosin (**13**), derived from *Astragali* root, exhibits antiproliferative and antimetastatic activities in the luminal BC cell lines T-47D and MCF 7. The mechanisms of action include suppression of basic leucine zipper transcription factor ATF-like (BATF) and modulation of the expression of E-cadherin, N-cadherin, vimentin, CD147, matrix metallopeptidase (MMP)-2, MMP-9, and the long non-coding RNA (lncRNA) WDR7-7 [300,301]. Furthermore, in MCF 7 cells, puerarin (**12**) and calycosin (**13**) activate caspase-3 [228,229]. Regarding the estrogenic pathway, a significant increase in the expression of ERβ was observed in MCF 7 cells after treatment with calycosin. This effect was associated with a reduction in IGF-1R, the activation of PARP-1, and the downregulation of miR-375. In that same study, the researchers noted that calycosin is more promising than formononetin [302]. Again, there are no ongoing or completed clinical trials with calycosin.

Finally, equol (**14**), also found in soybeans, has been associated with increased effectiveness of tamoxifen, which suggests a possible combined treatment for luminal BC. The ability of equol to bind with high affinity to ERβ has also been reported, resulting in the inhibition of cell proliferation and induction of apoptosis [248]. However, this activity is under investigation due to the possible controversial activity of equol [303,304,305]. In vitro studies have shown that the translation factor eIF4G is upregulated in cells that are treated with equol, resulting in increased translation of pro-oncogenic mRNAs, for example, the transcription factor c-Myc, which can consequently increase the viability of metastatic cells [306].

### 7.3. Alkaloids and Catechins

Alkaloids are a class of natural substances that have received considerable interest due to their therapeutic potential, including anti-inflammatory, antiviral, and antimicrobial activities. In cancer cells, they can trigger apoptosis and autophagy, reduce tumor size, inhibit cell proliferation, and can be used in combined therapeutic approaches [307,308]. Among the alkaloids, piperine is commonly cited for its antitumor activities in luminal BC.

**Figure 7:**
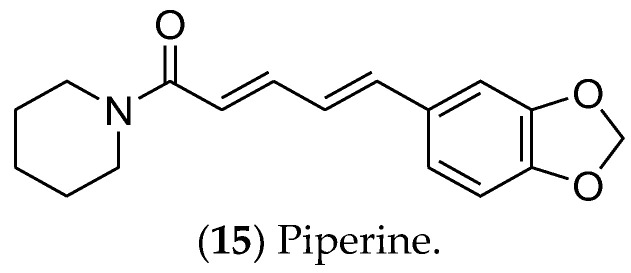
The alkaloid piperine, which has effects on luminal breast cancer.

Piperine (**15**) (Figure 7), an alkaloid obtained from black pepper (*Piper longum*), is an active compound in luminal BC cells [309]. It inhibits the Wnt/β-catenin, Hedgehog, and Notch pathways, which are involved in cancer stem cells’ self-renewal. In addition, piperine induces apoptosis and regulates the expression of proteins such as EGFR, VEGF, CDK, and NF-kβ [310]. Nanoparticles and liposomes have been used for piperine formulations with enhanced effectiveness, including reversion of multidrug resistance and sensitivity to paclitaxel and tamoxifen [310,311]. The mechanism of action by which piperine regulates the estrogen pathway remains unclear, given the lack of in vitro and in vivo assays focused on hormonal signaling.

**Figure 8:**
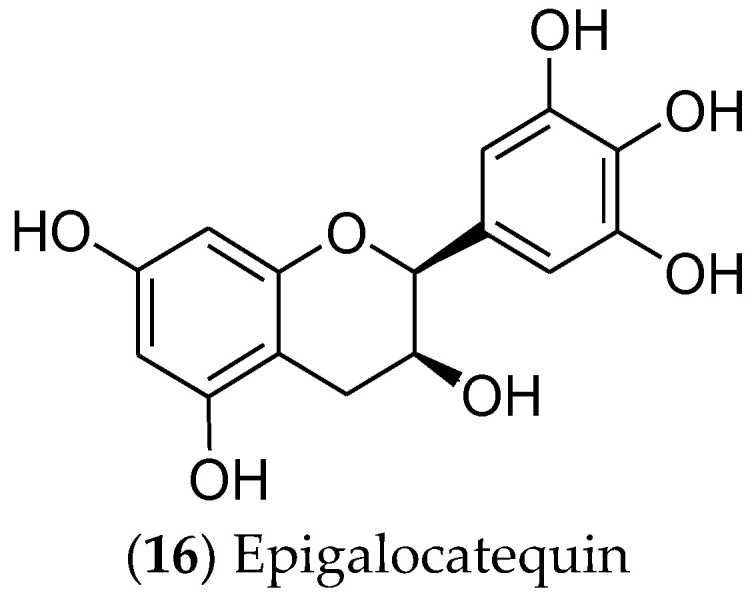
The catechin epigalocatequin, which has effects on luminal breast cancer.

In turn, catechins are polyphenolic phytonutrients that are found in green tea (*Camellia sinensis*). Among the catechins, epigallocatechin (**16**) [312,313] (Figure 8) is cytotoxic and selective for MCF 7 cells, regulating the EGFR, STAT3, ERK, ERK1/2, NF-κB, and Akt pathways [314,315,316,317]. Moreover, this compound is currently in phase I clinical trials for the prevention and treatment of radiodermatitis in patients with BC (ClinicalTrials.gov identifier: NCT01481818) [318].

### 7.4. Lignans

**Figure 9:**
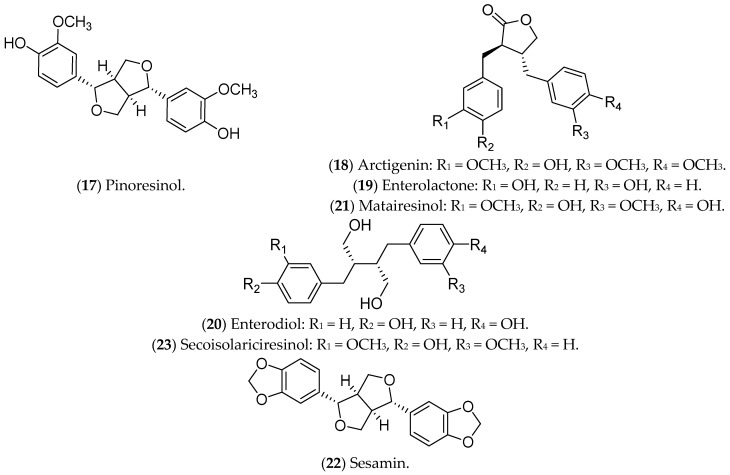
Lignans with effects on luminal breast cancer.

Lignans are phytoestrogens that are absorbed from plant sources and are associated with lower risks of postmenopausal BC, with promised effects against luminal BC [319]. Their main mechanism of action includes inhibition of NF-κB [320,321], with special attention devoted to pinoresinol, arctigenin, enterolactone, enterodiol, matairesinol, sesamin, and secoisolariciresinol (Figure 9).

Pinoresinol (**17**), a lignan commonly found in olives, binds to ERs, being selective and cytotoxic to luminal BC cells, with a pro-oxidant action [232]. Furthermore, pinoresinol selectively inhibits cell proliferation, induces apoptosis, blocks HER2 receptors, and increases ROS production [232,322]. Regarding the estrogenic pathway, it was previously demonstrated that pinoresinol apparently increases the viability of MCF 7 cells, with an ERα agonist action [323]. Another study, also conducted in vitro, demonstrated that the antiproliferative and cytotoxic action of pinoresinol is independent of the estrogen pathway when low concentrations of the compound are used. Therefore, pinoresinol’s activity in luminal BC is poorly understood and limited to in vitro assays [232].

Arctigenin (**18**), a bioactive compound from *Arctium lappa* L., also binds to ERs and reduces pro-tumor signals, such as granulocyte-macrophage colony-stimulating factor (GM-CSF), MMP-3, MMP-9, and thymic stromal lymphopoietin (TSLP). In addition, it inhibits the proliferation and invasion of luminal BC cells [233,324,325,326].

Some lignans are converted in the intestine into estrogenic enterolignans such as enterolactone (**19**) and enterodiol (**20**). Although both act through ERs, the mechanisms are different. Enterodiol binds ERα via the N-terminal activation domains 1 (AF-1) and 2 (AF-2) of the receptor, like ETD. Enterolactone, in turn, acts mainly via AF-2. Both compounds, however, affect the proliferation of MCF 7 cells [327]. Furthermore, enterolactone, in the presence of ETD, reduces the proliferation of MCF 7 cells, possibly modulating the hormonal effects [328]. Mali et al. (2012) showed that enterolactone downregulates the expression of MMP2, MMP9, and MMP14 and inhibits the adhesion, invasion, and migration of MCF 7 cells [329]. Along with enterolactone, enterodiol has also been evaluated for its antitumor activity against the proliferation of MCF 7 cells [330]. In addition, in vitro (MCF 7 lineage) and in vivo treatments with enterodiol and enterolactone inhibited hormone-induced tumor growth, even controlling the production of VEGF and angiogenesis [331]. This information highlights the potential of these compounds in the prevention and treatment of luminal BC, including some clinical trials that have already been carried out [332].

Matairesinol (**21**), found in seeds, vegetables, and fruits, has antiangiogenic, antitumor, and antifungal activities [333]. Abarzua and collaborators (2012) demonstrated the antitumor potential of matairesinol in MCF 7 cells, with a significant reduction in cell viability. However, the effects of matairesinol did not surpass those of enterolactone (**19**) and enterodiol (**20**) [236].

Sesamin (**22**), a phytochemical identified in *Sesamum indicum*, is metabolized by the liver and induces G1 cell-cycle arrest in MCF 7 cells [334], regulates ER and programmed death-ligand 1 (PD-L1) expression, and inhibits growth factors and tyrosine kinase pathways [238,335,336]. In murine models, sesamin reduced the expression of HER2 and VEGF, and it inhibited the MAPK signaling pathway [238,336]. The above results were limited to in vitro and in vivo tests in animal models.

Also, secoisolariciresinol (**23**), a compound extracted from seeds of *Linum usitatissimum*, modulates inflammation through the NF-kB pathway in MCF 7 cells [239], and it also alters the expression of *ER1*, *ER2*, *EGF*, *BCL2*, and *IGF1R* [240]. Moreover, secoisolariciresinol and its derivatives can also induce apoptosis in MCF 7 cells and potentiate the action of chemotherapeutic agents such as doxorubicin, being considered safe and tolerable in phase IIB studies [337]. These data demonstrate the potential of this compound for the treatment of luminal BC.

### 7.5. Coumestans and Stilbenes

**Figure 10:**
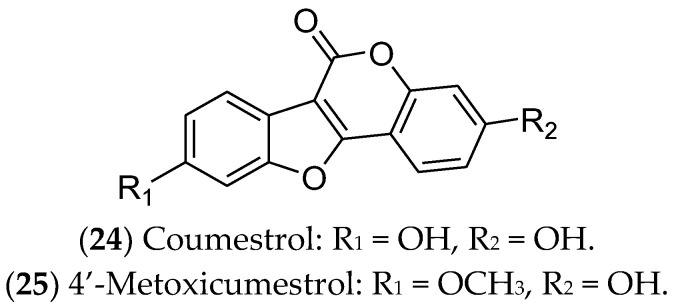
Coumestans with effects on luminal breast cancer.

Coumestans are polycyclic aromatic compounds that have a heterocyclic structure with four oxygenated rings, including coumarin and benzofuran moieties, connected by a carbon–carbon double bond. They exhibit biological effects similar to those of phytoestrogens and polyphenols, showing in vitro anticancer potential. However, in vivo studies using these compounds are still limited [338,339,340,341]. Among the coumestans with antitumor activity, we highlight coumestrol and 4′-methoxycumestrol, which have previously shown activity against luminal BC (Figure 10). Coumestans are mainly produced during the germination of beans, clovers, Brussels sprouts, and soybeans. The amounts of coumestans in plants may vary depending on the variety, growth stage, presence of diseases, location, and use of fungicides and insecticides [342].

Coumestrol (**24**) has already been identified in soybeans, clover, and spinach. It inhibits 17β-HSD enzymes and aromatase and binds to ERα and ERβ. In this sense, coumestrol regulates the hormone receptor pathways and expression, with anti-estrogenic activity 30–100 times greater than that of isoflavones. Therefore, this compound can be used as a complementary strategy in hormone therapy and chemotherapy for luminal BC [343,344,345,346]. In ER-positive cells, in addition to reducing cell viability, coumestrol significantly reduces the expression of genes that drive epithelial-to-mesenchymal transition (Snail), bone fixation (CXCR4 and integrin αV), and osteolysis (PTHrP and TNF-α) [344] Likewise, 4′-metoxicumestrol (**25**) is also cited as a phytoestrogen with an antiproliferative effect on luminal BC cells, downregulating Akt phosphorylation [242].

**Figure 11:**
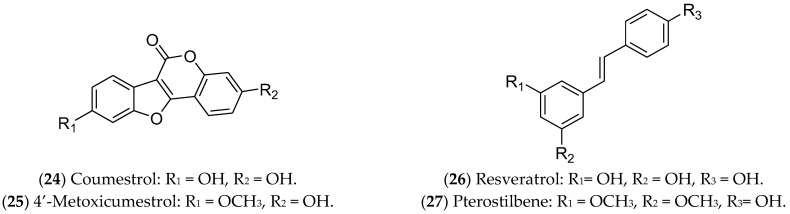
Stilbenes with effects on luminal breast cancer.

Stilbenes are natural substances isolated from vines, sorghum, pine, fir, and mulberry. These compounds have a core structure of 1,2-diphenylethylene and are used by plants as a defense against external threats, including pests, microorganisms, and the harmful effects of ultraviolet radiation [347]. Among the stilbenes, resveratrol (**26**) and pterostilbene (**27**) have been reported as active agents for luminal BC cells (Figure 11).

Resveratrol (**26**), also known as 3,5,4′-trihydroxy-trans-stilbene, is one of the most famous polyphenols and phytoestrogens, found mainly in grape skins, especially those of red grapes. Additionally, it can be found in blueberries, raspberries, cranberries, blackberries, peanuts, and cocoa powder [348]. This compound inhibits cell proliferation and reduces the migration and viability of BC cells [243,244]. Moreover, it exhibits synergistic effects when combined with chemotherapy agents such as doxorubicin, cisplatin, docetaxel, and paclitaxel [349]. Resveratrol triggers apoptosis in MCF 7 cells [350], arrests the cell cycle in the S phase [351], and causes DNA damage [352] and epigenetic alterations, such as in genomic methylation and miRNA expression [353]. Regarding the estrogen pathway, the compound is characterized as a weak agonist/antagonist of both ERs, being structurally similar to ETD [348,354]. Clinical trials indicated that resveratrol was safe and well tolerated, in addition to its action as a chemopreventive agent for BC patients [355].

Pterostilbene (**27**) is an analogue of resveratrol, found mainly in blueberries, and it also demonstrates antitumor activity against MCF 7 cells. Previous studies demonstrated that pterostilbene functions as an ERα inhibitor, while also inducing apoptosis [243,245,356]. Pterostilbene can induce apoptosis in mammary tumor cells by antagonizing ETD and specifically inhibiting ERα36 [357]. In addition, this stilbene can lead to an accumulation of neutral lipids in the intracellular environment, activating autophagy, reducing mitosis and metastasis [358], blocking the cell cycle, inducing morphological alterations and DNA degradation, increasing caspase-9 expression, and modulating the Akt/mTOR pathway [359,360].

### 7.6. Other Compounds

**Figure 12:**
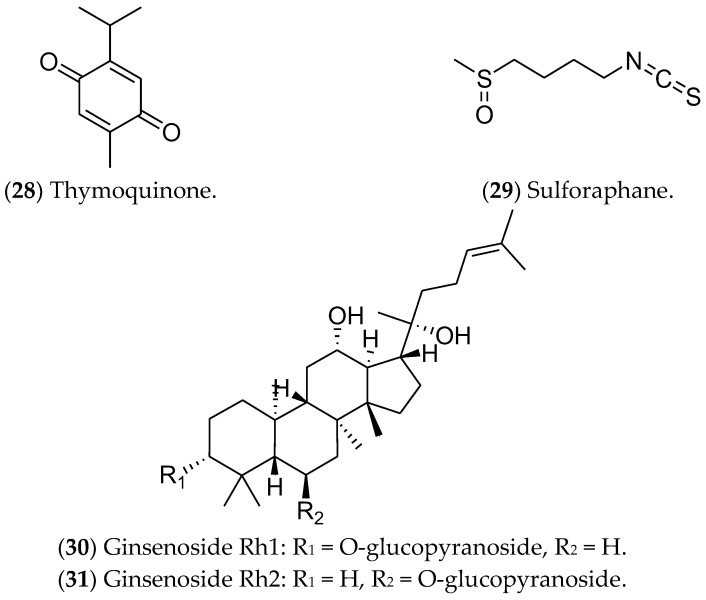
Other compounds with effects on luminal breast cancer.

Other compounds such as thymoquinone, sulforaphane, and ginsenosides have been studied as potentially active in luminal BC (Figure 12). Thymoquinone (**28**), a monoterpene found in *Nigella sativa*, can induce apoptosis via p53 in MCF 7 cells [361]. In addition, this compound can modulate NF-κB levels, arrest the cell cycle in the S phase [361], and alter the expression of genes related to the estrogen pathway [362]. Isothiocyanate sulforaphane (**29**) is found in broccoli, especially broccoli sprouts, and in cruciferous vegetables such as cabbage, cauliflower, and kale [363]; it decreases *ER1* expression [364]. This compound is found in cruciferous vegetables and regulates gene expression through epigenetics, inhibiting histone deacetylase (HDAC) [365]. Furthermore, sulforaphane inhibits cell proliferation, induces apoptosis, and arrests the cell cycle at the G2/M phase in MCF 7 cells [366]. Recently, a phase II clinical study found that the effect of a broccoli sprout preparation, in which sulforaphane is a key component, could increase the levels of protective enzymes in BC tissues (ClinicalTrials.gov Identifier: NCT00982319) [367]. 

Finally, ginsenosides (**30**, **31**), present in ginseng root (*Panax*), have been considered as possible antitumor agents for triggering apoptosis [368]. Huynh et al. (2021) showed high cytotoxicity of these compounds in MCF 7 cells, as well as increases in apoptosis, autophagy, and cell-cycle arrest. Furthermore, the ROS production after the treatments inhibited the PI3K/Akt pathway [249,250,369]. Despite showing antitumor action during in vitro tests, the use of these compounds in clinical trials is limited by the scarcity of data related to their metabolic regulation and modulated pathways [370].

## 8. The Challenges of Clinical Practice

Historically, natural products have contributed decisively to the treatment of tumors by inhibiting proliferation, metastasis, and angiogenesis, in addition to sensitizing transformed cells to radiotherapy and chemotherapy [371]. Synergism between compounds has also been explored, as it allows for increasing efficacy, reducing the administered dose, avoiding toxicity, and minimizing drug resistance [372]. However, natural products have low stability, poor absorption and biodistribution, and fast metabolism and excretion profiles [373].They are poorly soluble in water, with low lipophilicity and inappropriate molecular size. Furthermore, cellular transport and uptake are also low and, in this context, even when high doses are administered, concentrations in plasma and tissue are reduced [374]. Therefore, structural modifications are necessary to improve their bioavailability. For example, *trans*-2,4,3′,4′,5′-pentamethoxystilbene, a resveratrol derivative, showed higher potency and antiproliferative activity in MCF 7 cells [375]. Finally, nanotechnology has presented the resources to overcome these barriers by allowing for the delivery of encapsulated agents in an optimized therapeutic system. In this context, in a study conducted by Gadag et al. [376], nanostructured lipid carriers (NLCs) containing resveratrol enhanced its cytotoxicity against BC cells compared to pure resveratrol, along with increased permeation into the skin and bioavailability by oral administration. Despite being revolutionary, nanotechnology still faces regulatory aspects related to safety/toxicity, which reaffirm the challenges faced in the clinical use of natural products. The most promising compounds for the treatment of ER-positive BC, along with their clinical limitations, are described in Table 2.

## 9. Conclusions

**Figure 13:**
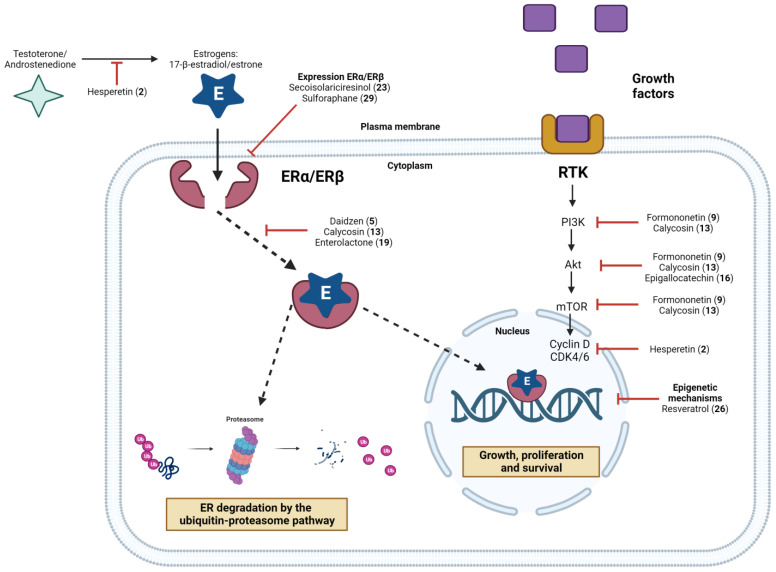
Scheme showing potential natural compounds for the treatment of ER-positive breast cancer by acting on estrogen signaling: E (estrogen), ERα/ERβ (estrogen receptors α and β), RTK (receptor tyrosine kinase), PI3K (phosphatidylinositol 3-kinase), Akt (protein kinase B), mTOR (mammalian target of rapamycin), CDK4/6 (cyclin-dependent kinases 4 and 6), SERMs (selective ER modulators), SERDs (selective ER downregulators), AIs (aromatase inhibitors). Created with BioRender.com. Accessed on 23 September 2023.

BC is molecularly heterogeneous and has challenged clinical practice. Estrogens play a critical role in the development of normal breast cells, but they contribute to the genesis and progression of tumors. Thus, luminal BC (ER-positive) is eligible for endocrine therapy, which has been widely used and is responsible for a significant increase in patient survival. However, with drugs in clinical routine, cases of resistance associated with relapse are of concern to oncologists and researchers. Secondary metabolites of plant origin have been a promising alternative in the search for new drugs, since they can modulate estrogenic signaling. Moreover, they can be structurally modified, can be incorporated in nanoformulations, and can be used as a treatment system alone or combined with currently used drugs. The natural compounds described in this review highlight the importance of some potentially pharmacologically exploitable plant species in the treatment of luminal BC. We highlight some of these compounds in Figure 13. Therefore, given the scarcity of clinical trials, the need for more detailed studies dedicated to unveiling the potential of these compounds in reversing resistance to hormone therapy is still evident. In fact, natural products can overcome barriers to optimized and innovative healthcare, meeting Sustainable Development Goals (SDGs) 03 and 08 of the WHO.

## Figures and Tables

**Table 1:** Natural compounds with effects on luminal breast cancer cell lineages.

Class	Compound	Main Sources	Cell Lineage	Mechanism of Action	References
Flavonoid	Hesperidin	Orange (*Citrus × sinensis*)	MCF 7	Induction of cell-cycle arrest in the G1 phase Inhibition of cell proliferation Induction of apoptosis	[213]
	Hesperetin	Orange (*Citrus × sinensis*)	MCF 7	Induction of cell-cycle arrest in the G1 phase Inhibition of cell proliferation Induction of apoptosis	[214]
	Luteolin	Algaroba (*Prosopis juliflora*)	MCF 7	Inhibition of IGF-1 stimulation by the PI3K-Akt signal transduction pathway	[215,216]
	Apigenin	Chamomile (*Matricaria recutita* L.)	MCF 7	Phosphotransferase inhibition	[217,218]
Isoflavonoid	Daidzein	Soy (*Glycine max* (L.) Merril)	MCF 7	Inhibition of CYP1 Induction of apoptosis Inhibition of topoisomerase Inhibition of cell-cycle arrest in G1 and G2 Phosphotransferase inhibition Activation via PI3K/Akt Inhibition of hTERT expression Increased CDKI protein expression Decreased protein expression in cyclins A, B, E, CDK1, CDK2, CDK4, CDK6, p21, p57, and p27	[219,220,221]
	Genistein	Soy (*Glycine max* (L.) Merril)	MCF 7	Inhibition of CYP1 Inhibition of DNMT1 Induction of apoptosis Inhibition of NF-κB activation Inhibition of telomerase and topoisomerase Phosphotransferase inhibition Activation via PI3K/Akt Inhibition of hTERT expression Increased p53 protein expression	[222,223,224]
	Glycitein	Soy (*Glycine max* (L.) Merril)	MCF 7	Inhibition of cell-cycle arrest in G1 and G2, and decrease in glucose uptake	[221,225]
	Biochanin A	Soy (*Glycine max*)	MCF 7	Cell-cycle arrest induced by upregulation of Bcl-2 expression Inhibition of cell-cycle arrest in G1 and G2	[221,226]
	Formononetin	Red propolis	MCF 7	Induction of cell-cycle arrest by the IGF-1/IGF-1R, MAPK, and PI3K/Akt signaling pathways Inhibition of hTERT expression Decreased mRNA and protein expression of D1 cyclins	[227]
	Glabridin	Licorice (*Glycyrrhiza inflata*)	MCF 7	CK activation in estrogen-responsive tissues	[221]
	Glabrene	Licorice (*Glycyrrhiza glabra*)	MCF 7	CK activation in estrogen-responsive tissues	[221]
	Puerarin	Kudzu (*Pueraria montana*)	MCF 7	Activation via PI3K/Akt	[228]
	Calycosin	Red propolis	MCF 7	Induction of apoptosis	[229]
	Equol	Soy (*Glycine max*)	MCF 7	Induction of apoptosis	
Alkaloid	Piperine	Black pepper (*Piper nigrum* L.)	MCF 7	Antiproliferative effect Induction of apoptosis Activation of caspase-3 and PARP cleavage Inhibited expression of the *HER2* gene at the transcriptional level Blocked ERK1/2 signaling to reduce SREBP-1 expression Inhibition of AP-1 activation	[230]
Catechin	Epigallocatechin	Green tea (*Camellia sinensis*)	MCF 7	Induction of apoptosis	[231]
Lignan	Pinoresinol	Indian tea (*Camellia sinensis*)	MCF 7	HER2 protein proteasomal degradation Induction of apoptosis	[232]
	Arctigenin	Burdock (*Arctium lappa* L.)	MCF 7	Downregulation of cyclin D1 protein expression	[233,234]
	Enterolactone	Linseed (*Linum usitatissimum* L.)	MCF 7	Downregulation of FAK/paxillin pathway phosphorylation	[235]
	Matairesinol	Linum (*Linum sp*)	MCF 7	Downregulation of the ER-β receptor, cutting off the G0 and G1 mitotic phase	[236]
	Enterodiol	Linseed (*Linum usitatissimum* L.)	MCF 7	Inhibition of VEGF secretion	[237]
	Sesamin	Sesame (*Sesamum indicum* L.)	MCF 7	Negative activation of EGFR and MAPK expression	[238]
	Secoisolariciresinol	Burdock (*Arctium lappa* L.)	MCF 7	Inhibition of NF-kB Phosphotransferase inhibition Inhibition of CYP1 Activation via PI3K/Akt Decreased expression of CDK6	[239,240]
Coumestan	Coumestrol	Alfalfa (*Medicago sativa* L.)	MCF 7	Inhibition of CK-2 phosphotransferase activity Inhibition of hTERT expression Decreased protein expression in cyclin E and CK-2	[220,241]
	4-Methoxycoumestrol	Soy (*Glycine max*)	MCF 7	Downregulation of CK-2-specific Akt phosphorylation	[242]
Stilbenoid	Resveratrol	Blueberry (*Vaccinium* spp.) and Blackberry (*Morus* spp.)	MCF 7	Inhibition of CYP-1A1/1A2/1B1 and 2E1 Decreased protein expression of cyclin D1	[243,244]
	Pterostilbene	Blueberry (*Vaccinium* spp.)	MCF 7	Induction of apoptosis Decreased mRNA and protein expression of D1 cyclins	[243,245]
Monoterpene	Thymoquinone	Black cumin (*Nigella sativa* L.)	MCF 7 e T-47D	Activation of caspases 8, 9, and 7 Increased PPAR-γ activity and Bcl-2/Bcl-xL expression Inhibition of the PI3K/Akt pathway and induction of p53 and p21 protein expression	[246,247]
Isotiocianate	Sulforaphane	Broccoli (*Brassica oleracea*)	MCF 7 e T-47D	Interruption of proliferation and mitosis Inhibition of ER-α protein expression B1 cyclin elevation Decreased EGFR, HER2, and hTERT mRNA expression	[248]
Saponin	Ginsenoside Rh1	Ginseng (*Panax ginseng*)	MCF 7	Induction of apoptosis	[249]
Tab	Ginsenoside Rh2	Ginseng (*Panax ginseng*)	MCF 7 e ADM	Reverses P-gp-mediated drug resistance of MCF 7/ADM cells	[250]

**Table 2:** Promising natural compounds for the treatment of ER-positive breast cancer, and challenges for their clinical application.

Natural Compound	Potential Targets and Mechanisms of Action in the Context of Estrogen Signaling	Preclinical and Clinical Evaluation in the Context of BC	Challenges for Its Use in Clinical Practice	Reference
Hesperetin (2)	- Regulates estrogen metabolism, and induces both extrinsic and intrinsic apoptotic pathways - Suppresses aromatase enzyme activity and cyclin D1, CDK4, Bcl-xL, and pS2 expression	- Reduced the tumor growth in female athymic mice with BC	- Possibly toxic to the liver - Recommended long-term animal and clinical studies to understand its therapeutic advantages in cancer	[257]
Daidzein (5)	- Regulates estrogen and estrogen receptor complex-binding affinity - At high concentrations, exhibits anticancer capacity	- Phase I multiple-dose clinical investigation to test the safety and effects in healthy postmenopausal women (ClinicalTrials.gov Identifier: NCT00491595)	- The mechanisms of action are still not completely known, and its poor bioavailability restricts its clinical application - Possibly causes unwanted side effects	[273,377]
Formononetin (9)	- Induces cell-cycle arrest in BC cells via IGF1/PI3K/Akt pathways	- Showed growth-inhibitory activity associated with inhibition of tumor angiogenesis in xenograft models of BC	- There is still insufficient evidence to delineate the exact anticancer mechanisms	[287,288,378]
Calycosin (13)	- Inhibits growth and induces apoptosis in ER-positive BC cells via ERβ-dependent regulation of the IGF-1R, p38 MAPK, and PI3K/Akt pathways	- Inhibited tumor growth in mice bearing MCF 7 or SKBR3 xenografts	- Recommended long-term animal and clinical studies to better understand its toxicity and therapeutic advantages in cancer	[301,379]
Epigallocatechin (16)	- Can exert cytotoxic effects in MCF 7 cells, possible through the EGFR, STAT3, ERK, ERK1/2, NF-κB, and Akt pathways	- Oral treatments in mice resulted in a reduction in tumor growth and antiangiogenic effects in xenograft and allograft models of BC - Evaluated in phase I clinical trials for the prevention and treatment of radiodermatitis in patients with BC (ClinicalTrials.gov Identifier: NCT01481818)	- Low oral bioavailability is a problem for its therapeutic application - There is still insufficient evidence about the molecular mechanisms involved in its protective effects against mammary carcinogenesis - More in vivo studies are necessary to determine its potential toxicity	[316,317,318]
Enterolactone (19)	- Indicated anti-estrogenic effects and affected VEGF production in ER-positive breast cancer	- Showed some benefit to BC patients’ prognosis when it was found at higher concentrations in the serum - Its lower concentration in the serum was associated with an increased risk of developing BC	- Long-term studies are needed to understand its potential benefits or harms to BC patients	[380,381]
Secoisolariciresinol (23)	- Alters the expression of ER1, ER2, EGF, BCL2 747, and IGF1R	- Phase II clinical studies were conducted in premenopausal women at risk of developing breast cancer, but they did not indicate significant results in Ki-67 expression compared to the placebo-treated group - The trials demonstrated that its use is tolerable and safe	- More clinical trials are necessary to determine its real potential for treating BC patients	[240,337]
Resveratrol (26)	- Reduces the expression of certain breast-cancer-related genes (e.g., RASSF-1α) via epigenetic mechanisms	- Inhibited the growth of Erβ-positive tumor explants, increased apoptosis, and decreased angiogenesis in nude mice - Clinical trials showed that resveratrol was safe and well tolerated, in addition to its action as a chemopreventive agent for BC patients	- The major obstacle presented in the clinical trials was its poor bioavailability	[382,383]
Sulforaphane (29)	- Can inhibit the expression of ERα protein in MCF 7 cells, affecting its mRNA levels or mediating the degradation of the receptor by the proteasome complex - Suppresses MCF 7 cell growth via the miR-19/PTEN axis	- A phase II clinical study examined whether this compound in a broccoli sprout preparation could increase the levels of protective enzymes in BC tissues (ClinicalTrials.gov Identifier: NCT00982319)	- Available on the market as a food supplement - More efforts are necessary to determine its therapeutic properties in BC patients	[364,366,367,384]

## Data Availability

Not applicable.
